# Effects of Lifestyle Interventions That Include a Physical Activity Component in Class II and III Obese Individuals: A Systematic Review and Meta-Analysis

**DOI:** 10.1371/journal.pone.0119017

**Published:** 2015-04-01

**Authors:** Aurélie Baillot, Ahmed J. Romain, Katherine Boisvert-Vigneault, Mélisa Audet, Jean Patrice Baillargeon, Isabelle J. Dionne, Louis Valiquette, Claire Nour Abou Chakra, Antoine Avignon, Marie-France Langlois

**Affiliations:** 1 Research Center of the Centre hospitalier universitaire de Sherbrooke, Sherbrooke, Quebec, Canada; 2 Department of Medicine, Division of Endocrinology, Université de Sherbrooke, Sherbrooke, Quebec, Canada; 3 Unit of Nutrition and Diabetes, Department of Endocrinology-Nutrition and Diabetes, University Hospital of Montpellier, Montpellier, France; 4 Research Centre on Aging, Health and Social Services Centre, Institute of Geriatrics, Université de Sherbrooke, Sherbrooke, Quebec, Canada; 5 Faculty of Physical Education and Sports, Université de Sherbrooke, Sherbrooke, Quebec, Canada; 6 Department of Microbiology and Infectious Diseases, Université de Sherbrooke, Quebec, Canada; 7 INSERM U1046, Physiology and experimental medicine of heart and muscles, University of Montpellier, Montpellier, France; Medical University Innsbruck, AUSTRIA

## Abstract

**Background:**

In class II and III obese individuals, lifestyle intervention is the first step to achieve weight loss and treat obesity-related comorbidities before considering bariatric surgery. A systematic review, meta-analysis, and meta-regression were performed to assess the impact of lifestyle interventions incorporating a physical activity (PA) component on health outcomes of class II and III obese individuals.

**Methods:**

An electronic search was conducted in 4 databases (Medline, Scopus, CINAHL and Sportdiscus). Two independent investigators selected original studies assessing the impact of lifestyle interventions with PA components on anthropometric parameters, cardiometabolic risk factors (fat mass, blood pressure, lipid and glucose metabolism), behaviour modification (PA and nutritional changes), and quality of life in adults with body mass index (BMI) ≥ 35 kg/m^2^. Estimates were pooled using a random-effect model (DerSimonian and Laird method). Heterogeneity between studies was assessed by the Cochran’s chi-square test and quantified through an estimation of the *I* ^²^.

**Results:**

Of the 3,170 identified articles, 56 met our eligibility criteria, with a large majority of uncontrolled studies (80%). The meta-analysis based on uncontrolled studies showed significant heterogeneity among all included studies. The pooled mean difference in weight loss was 8.9 kg (95% CI, 10.2–7.7; p < 0.01) and 2.8 kg/m^²^ in BMI loss (95% CI, 3.4–2.2; p < 0.01). Long-term interventions produced superior weight loss (11.3 kg) compared to short-term (7.2 kg) and intermediate-term (8.0 kg) interventions. A significant global effect of lifestyle intervention on fat mass, waist circumference, blood pressure, total cholesterol, LDL-C, triglycerides and fasting insulin was found (p<0.01), without significant effect on HDL-C and fasting blood glucose.

**Conclusions:**

Lifestyle interventions incorporating a PA component can improve weight and various cardiometabolic risk factors in class II and III obese individuals. However, further high quality trials are needed to confirm this evidence, especially beyond weight loss.

## Introduction

Obesity is now recognized as the most prevalent metabolic disease world-wide, reaching epidemic proportions in both developed and developing countries [1]. In North America, the prevalence of class II and III obesity (Body mass index (BMI) ≥35 kg/m²) has increased rapidly over the last decade [2,3]. Severe obesity is associated with multiple comorbidities such as hypertension, insulin resistance, type 2 diabetes, dyslipidemia, cardiovascular disease, sleep apnea and cancer [4,5], and is often associated with musculoskeletal pain [6,7]. All these comorbidities further lead to impaired health-related quality of life [8,9]. The importance of obesity is also obvious when looking at the considerable resources dedicated to its treatment and care, which account for between 0.7% and 2.8% of a country's total healthcare expenditures [10].

Several strategies are recommended for the treatment of obesity, including dietary therapy, regular physical activity (PA), behavioral therapy (BT), pharmacotherapy, and bariatric surgery as well as combinations of these strategies [11–15]. Although, bariatric surgery remains the most effective treatment to decrease and maintain weight loss, as well as improve comorbidities and mortality [16,17], lifestyle intervention is recommended as the first step to achieve weight loss and to treat obesity-related comorbidities in subjects with severe obesity [12]. In addition, given the limited resources, lifestyle intervention remains an effective option to help more subjects with severe obesity [18] and subjects could also prefer less invasive treatment than bariatric surgery [19].

PA is an important component of lifestyle intervention and should be systematically included in lifestyle management components [12]. PA, self-monitoring, and continued follow-up contacts have been identified as key components of weight control [20]. In addition, several studies showed that PA presents several benefits in individuals with class II and III obesity [21], as well as in class I: improvement of morbidities, cardiovascular diseases mortality and quality of life [21–25]. However, non-surgical obesity programs in Canada include less PA support compared to nutritional support (73 vs. 93%) and have less PA professionals compared to dietitians (43 vs. 74%) [26].

Previously, a review and meta-analysis of lifestyle interventions in obese and overweight individuals concluded that they can significantly reduce body weight and cardiometabolic risk factors in the mid- to long-term [27]. However, no systematic literature review is currently available on the effect of lifestyle interventions (dietary intervention, PA, BT) specifically in class II and III obese individuals. Thus, the present systematic review aims to give an overview of lifestyle interventions that include a PA component (counseling, recommendations, education or exercise training) proposed to more severe obese individuals. We thus carried out a systematic review, meta-analysis and meta-regression on the effects of lifestyle interventions incorporating a PA component among class II and III obese on i) anthropometric parameters; ii) cardiometabolic risk factors; iii) behaviour modification; iv) and quality of life. The secondary objectives were i) to investigate the impact of sex, age, severity of obesity and metabolic disorders on the lifestyle interventions efficiency; ii) to compare lifestyle intervention modalities; and iii) to assess the long-term impact of lifestyle intervention in this population.

## Methods

### Information sources and study selection

This systematic review followed the guidelines of the Preferred Reporting Items for Systematic Reviews and Meta-analysis (PRISMA) [28]. The information sources and study selection methods were described elsewhere [21]. Briefly, the research was completed on November 16^th^, 2012 across 4 databases (Medline, Scopus, CINAHL and Sportdiscus) using specific keywords and Medical Subject Headings [21]. Two independent reviewers screened all records according to titles and/or abstracts (AB and MMRF) and assessed selected full-text articles for inclusion and exclusion criteria (AB and MA). Disagreements were resolved by a third party (MFL) and reviewers’ agreement was calculated using Cohen’s kappa coefficient [29].

### Eligibility criteria

The following inclusion criteria were applied: i) peer-reviewed original studies; ii) class II and III obese adults (>18 years; more than 75% of the sample with BMI≥35 kg/m^2^ and no normal weight subject); iii) lifestyle interventions, incorporating a PA component (counselling, recommendations, education, or exercise training) and with at least one of these components: BT, diet, nutritional education or recommendations or counselling; and iv) at least one of these outcomes: anthropometric parameters (body weight, waist circumference), cardiometabolic risk factors (% of fat mass, lipid or glucose metabolism, blood pressure), PA or nutritional behaviors (energy expenditure or intake, recommended healthy behaviors), and quality of life.

BT was considered as an approach focusing on modifying the perception of the environment to increase stimuli that promote healthy eating and PA behaviours while decreasing stimuli that make healthy eating and exercise challenging [30]. Studies with interventions including jaw fixation, anti-obesity medication or bariatric surgery were not considered unless they included at least one lifestyle intervention arm and only patients enrolled in the arm of interest were considered. No language restriction was applied. Authors were contacted twice in case of missing or incomplete data for the study selection or when more details on the intervention effects or population were needed. When more than one publication studied the same cohort and had overlapping results, only the most recent was considered.

### Data collection process

One reviewer (AB) extracted data for each study: country, design, sample size, baseline subject characteristics, lifestyle intervention modalities [length (months), follow-up length (months), attendance (%), frequency of contacts (number of contacts per month), delivery mode (group, individual face to face or telephone), type and number of professionals involved)], lifestyle intervention components [material support (documentation, website, pedometer, log…), exercise training, unsupervised exercise program, PA recommendations, caloric restriction, nutritional recommendations or education, BT]. The frequency of contacts was categorized as low (<1 session/month), moderate (1 or 2 sessions/ month), and high (>2 sessions/month) or missing information [15]. Studies were categorized as exercise training only if a professional totally or partially supervised the exercise sessions. Non-supervised exercise programs corresponded to interventions with individualized exercise plans without supervision. PA recommendations category corresponds to interventions that provide only general PA advices or studies that did not provide enough details about the intervention. Means with standard deviation (SD) of baseline, different evaluation time, post-intervention and follow-up outcomes of interest and p-values were reported. Missing baseline characteristic for subgroups or final sample were replaced by the initial whole baseline population characteristics means. Only results from intention-to-treat analyses were extracted, when completers’ data were also available. All extracted data were double checked by another reviewer (KBV). Disagreements were resolved by a third party (MFL).

### Quality assessment in individual studies

The quality assessment of the included articles was performed (AB) using the Quality Assessment Tool for Quantitative Studies developed by the Effective Public Health Practice Project [31], as reported previously [21]. A second independent investigator (MA) conducted quality control of one third of randomly selected articles (n = 19). We reassigned study design according to the data used in our analysis. For example, if a study randomized subjects in lifestyle intervention with or without jaw fixation, we considered the design as an uncontrolled clinical trial since we only used data of the control group.

### Statistical analyses

Although some of included studies were controlled studies, for the present meta-analysis there were not enough “true” control groups to pool only randomized controlled trials. For this reason, all study groups were included in a longitudinal meta-analysis. Studies of weak quality were not included in the meta-analysis [32], as well as studies with only follow-up results or missing result data [33–37]. Subgroups analyses were performed according to the intervention lengths: short-term (<6 months), intermediate-term (6–11.9 months) and long-term (≥12 months) [15]. Studies with variable length of intervention for each subject [38] and no exact length of intervention [39] were excluded from the subgroup analysis. Baseline data and post-intervention outcomes were reported as absolute change in mean and the standard error of means (SEM). Associated SEM were calculated using SEM_diff._ = SEM_baseline_ + SEM_final—_2r SEM_baseline_ × SEM_final._ We assumed a moderately conservative coefficient of correlation r = 0.5.

Due to the heterogeneity among included studies, estimates were computed using a random-effect model with the DerSimonian and Laird method [40] that does not assume interventions to be similar and further includes inter-studies heterogeneity (Tau²) in the calculation [41].

Results are presented as mean difference with 95% confidence interval (CI). Heterogeneity between studies was assessed by the Cochran’s chi-square test (Q) and its extent was quantified through an estimation of the *I*². Expressed in percentage, the *I* ^2^ statistic describes the proportion of total variance in effect estimates due to the heterogeneity. Thus, homogeneous pooled studies should have an *I*² close to 0. Conventionally, 25%, 50%, and 75% respectively represent low, medium, and high inconsistency between studies [42,43].

In the presence of heterogeneity, meta-regressions with a random-effect model were performed to test different moderators available in the included studies and known to affect the final estimates such as sample size at inclusion, studies length in months, age of included participants and contact frequencies [44]. Data were analyzed with Open Meta-Analyst [45].

## Results

### Study selection

**Fig 1:**
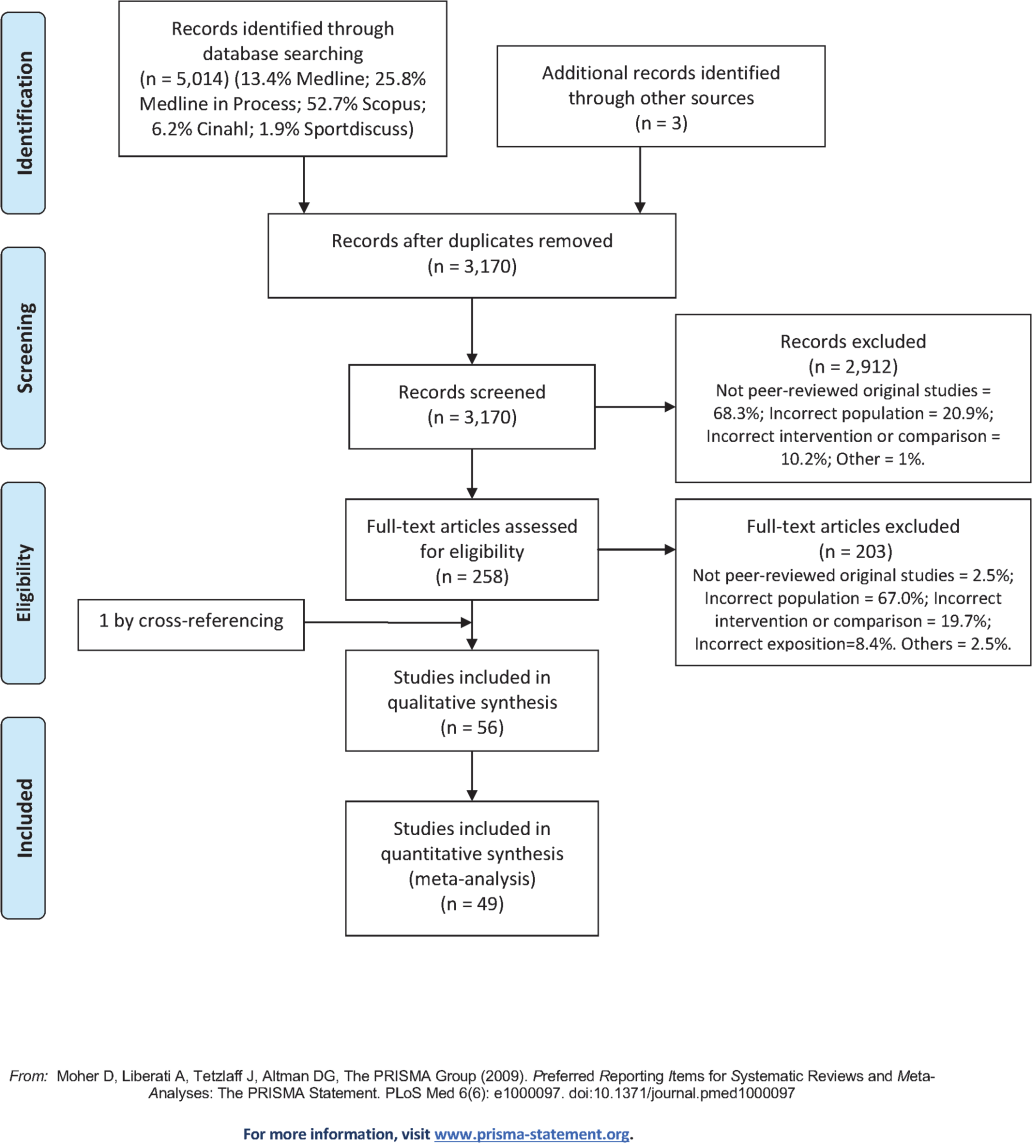
PRISMA 2009 Flow Diagram.

The electronic search identified 5014 publications, among which 56 articles were included in the review (Fig. 1). Reviewers had a moderate agreement score concerning screening title/abstracts (kappa coefficient = 0.7) and an excellent score for eligible studies (kappa coefficient = 0.93) [29]. Given that one article [46] provided data from a subpopulation (normal or abnormal glucose tolerance subjects) of a larger study [47], we did not consider this study in the calculation of percentage in the following part (100% = 55 articles), but kept it for the part effects of metabolic disorders on the effectiveness of lifestyle interventions.

### Study characteristics

Table 1 presents the characteristics and intervention modalities.

**Table 1:** Characteristics of controlled and uncontrolled studies from the longest to the shortest length of intervention in class II and III obese individuals (56 studies).

Ref. Design Country *Population specificity*	N by group (%W)	Age years± SD or (range)	BMI kg/m^2^±SD or (range)	Length of intervention (months)	Category of contact frequency	Intervention description	
Studies with controlled groups							
Bjorvell [49] CCT Sweden	15 (0)*	41.0±16.0	42.9 ±5	48	3	**Weight-loss phase (6 wk.):**	**Maintenance phase (48 months):**
						- Supervised exercise training in group or individual: 4x /wk.	- Weekly booster sessions or contacts by telephone/letter
						- Diet: 600 kcal/d.	- 2-wk periods of rehearsals at the ward to avoid relapse, if necessary
						- Behavioral treatment: 2 group contacts /wk.	
	3 (0)*	41.0±12.1	41.4 ±3.8	12	1	**Control program:**	
						- 2 interviews of 45 minutes	
						- Written program on reducing weight	
Richman [53] CCT Australia	39 (62)	45.6±10.6	47.3	12.8	Not provided	**Very low energy liquid diet:**	
						- Prescribed endurance exercise training: 3x/d.	
						- Diet: 412 kcal/d. within 3 provided meal replacements for 3 wk.; then 653 kcal/d within 0–2 meal replacements, as needed	
						- Behavior modification program: instructions on food, nutrition, exercise, stress management, relaxation techniques, methods to improve self-esteem coping strategies. Employed techniques included stimulus control, cognitive restructuring, and positive reinforcement.	
	23 (87)	45.2±11.0	47.4			**Standard kilojoule restriction:**	
						- Identical intervention except for the diet: 1200–1400 kcal/d for 12 mo.	
Goodpaster [55] RCT USA	67 (85)	46.1±6.5	43.7±5.9	12	3	**Initial PA:**	
						- Unsupervised moderate-intensity PA up to 5x 60 min /wk. with pedometer, diary and exercise videos	
						- Diet: 1200–2100 kcal/d. with liquid and prepackaged meal replacements provided	
						- Behavioral lifestyle intervention program: 4 group, individual or telephone contacts/month	
	63 (92)	47.5±6.2	43.5±4.8			**Delayed PA:**	
						- Identical intervention with 6-month delayed PA	
Martins [52] CCT *Prebariatric subjects* Norway	64 (58)	42.0±9.8	45.3±5.5	12	3	**Residential intermittent program (8–10 wk. center, 8 wk. home, 4 wk. center, 4–5 mo. home, 2 wk. center):**	
						- Structured and supervised PA: 5 group or individual sessions/wk.	
						- 6 meals/d. provided or prepared in groups under supervision	
						- Nutrition education program (energy needs and intake, healthy eating and cooking)	
						- Group-based psychotherapy to bring patients to be in charge of their lifestyle changes	
	30 (70)	38.4±10.1	48.3±6.6		3	**Commercial weight loss camp intensive intervention phase (5mo.):**	
						- Structured supervised PA: 5x120 min/wk. at 50–60% of VO_2_max.	
						- Diet: 2190 kcal/d. + daily educational nutrition	
						- Weekly cognitive strategies	
						**Home phase (7 months):**	
						- Bimonthly individual or telephone contacts to discuss dietary intake/patterns, PA level and behavioral modifications	
	57 (82)	41.4±9.9	44.3±5.3		3	**Hospital outpatient program weight loss phase (6 mo.):**	
						- Individualized, supervised and non-supervised PA: 3x/wk.	
						- Weekly group meetings on habits, nutrition or occupational therapy	
						**Maintenance phase (6 mo.):**	
						- Weekly PA group meetings + 3 motivation group meetings to keep lifestyle changes	
Annesi [58] RCT USA	183 (83)	42.5±10.0	41.7±6.5	6	2	**Standard nutrition education:**	
						- Individualized endurance PA up to 150 min/wk. of moderate intensity.	
						- PA: 6 individual 1h meetings on cognitive-behavioral methods to foster adherence in PA: goal settings, restructuring unproductive thoughts, addressing cues to exercise, preparedness for occurrences of barriers to exercise and relapse prevention	
						- Nutrition: 6 group sessions of 1h on nutrition: understanding healthy eating. 1) provision of information on consequences, and 2) general encouragement.	
	247 (83)					**Cognitive-behavioral methods for controlled eating:**	
						- Identical intervention except for nutrition: Cognitive-behavioral: additional array of behavior change techniques used in exercise support component.	
Parikh[50] RCT *Prebariatric subjects* USA	29 (90)	44.1±12.1	46.3±5.5	6	2	**Medically supervised weight management:**	
						- 5 monthly group class on dietary and PA education to promote health and weight loss, and individualized behavior modification counseling and goal-setting for weight loss.	
	26 (77)	46.2±12.7	44.7±7.1		1	**Usual care:**	
						- ≥ 1 visit for counseling at the clinical center	
Hemmingsson [48] RCT Sweden	20 (79)	43.0±12.6	43.8±5.2	4.5	2	**Standard support:**	
						- PA prescription up to 10 000 steps/d with pedometer and PA booklet	
						- 2-h group sessions every month offering support to increase PA, dietary changes, body weight diaries, home assignments (cooking, stress management, rewards, relapse prevention), and working on reinforcing positive aspects, boosting self-efficacy and increasing autonomy	
	22 (79)	43.9±13.3	40.1±5.3		3	**Added support:**	
						- Identical intervention with 10 additional walking promotion group meetings of 2 h.	
Reis [51] RCT *Prebariatric subjects* Brazil	10 (0)	36.7±11.5	55.7±7.8	4	Not provided	**Lifestyle intervention:**	
						- Supervised exercise training: 5x30 min/wk. of moderate exercise	
						- Individualized low-energy diet	
	10 (0)	42.2±11	54.0±6.1			**Usual care:**	
						- General, oral, and written information about healthy food choices and general guidance to increase the PA level	
Lafortuna [54] RCT Italy	15 (60)	33.5±7.8	40.4±3.3	0.75	3	**Individualized exercise training:**	
						- Supervised endurance and resistance training: 5x35 min/wk at 50–60% VO_2_max; 5x(1x15 repetitions)/wk. at 40–60% 1RM	
						- Diet: 1200–1800 kcal/d with daily group lectures, demonstrations, and discussions.	
						- 2–3 sessions/wk. of individual or cognitive-behavioral strategies: stimulus control procedures, problem solving, stress management skills, goal setting, development of healthy eating habits, assertiveness training, facilitation of social group supports, cognitive restructuring of negative maladaptive thoughts, relapse prevention training	
	15 (60)	34.3±11.0	40.0±4.7			**Non-specific exercise training:**	
						- Identical intervention except for PA: supervised endurance and resistance training: 5x30 min/wk at 30–45% VO_2_max+ leisure walking 2x50–70min/wk at 45–60% VO_2_max +5x30 min/wk. of body weight resistance training	
Sartorio [56] RCT Italy	52 (69)	34.0±8.0	41.3±5.1	0.75	3	**Baseline exercise training:**	
						- Non-individualized supervised endurance training: 5x60 min/wk.	
						- Diet: 1200–1800 kcal/d.	
						- Psychological counseling program: 2–3 individual contacts/wk. + daily group lectures, demonstrations, and discussions	
	22 (73)	29.0±7.0	42.2±5.7			**Endurance training:**	
						- Identical intervention except for PA: Supervised endurance training: 5x35 min/wk. at 50–60% VO_2_max.	
	22 (77)	30.0±8.0	41.5±4.2			**Endurance and strength training:**	
						- Identical intervention except for PA: Supervised endurance and resistance training: 5x30 min/wk. at 50–60% VO_2_max + 5x(1x15 repetitions)/wk. at 40–60% 5RM	
Sartorio [57] CCT Italy	26 (73)	29.8±7.9	41.1±4.1	0.75	3	**Endurance and strength training:**	
						- Idem, Lafortuna et al. [54]: group 1	
	26 (73)	29.1±6.6	41.7±5.3			**Endurance training:** Identical intervention except for PA: Individually supervised endurance training: 5x35 min/wk at 50–60% VO_2_max	
**Studies without controlled group**							
Golay [68] Switzerland	55 (82)	49.5±2.0	40.0±0.7	61.5	3	**Weight-loss phase (1.5 mo.):**	**Maintenance phase (60 mo.):**
						- Aerobic exercise training: 5x120 min/wk.	- Diet: 500 kcal deficit/d. supported by regular diet reviews
						- Diet: 1200 kcal/d	- PA activity recommendation: 1–2x30–60 min/d at 60% of HR max.
						- Nutritional education group and individual meetings: 2x/wk.	
						- 6 behavioral therapy sessions: self-control, cognitive restructuring reinforcement and relapse prevention.	
Anderson [38] USA	80 (69)	42.63	45.5±5.4	30.4	3	**Weight-loss phase (6.4 mo):**	**Maintenance phase (24 mo):**
						- Walking recommendation: 2000 kcal/wk.	- Daily records of food and PA calories
						- Diet: 520 kcal/d within ≥ 5 provided meal replacements	- 5 monthly group meetings or seminars. Periodic restaurant meals
						- Weekly behavioral education group classes focusing on acquiring skills to produce long-term weight maintenance and lifestyle changes	
Dixon [59] *OSA subjects* Australia	30 (40)	50.0±8.2	43.8±4.9	24	1	- Individualized structured moderate-intensity endurance and resistance training counting for 200 min/wk. + walking	
						- Diet: 500 kcal deficit /d, optional meal replacements as needed + dietary advice	
						- Individualized behavioral program (no more details available)	
Maffiuletti [69] Italy	64 (70)	30.2±7.2	41.3±4.3	12.8	3	**Weight-loss phase (0.75 mo.):**	
						- Supervised endurance and resistance training: 5x30–40 min/wk. at 40–70% of VO_2_max + 5x (1x15 repetitions)/wk. at 40–70% 1RM.	
						- Diet: 1200–1800kcal/d supported by 60-min daily nutritional education consisted of lectures, demonstrations and group discussions	
						- Psychological counseling: 2–3x60 min/wk. and based on individual or/and cognitive-behavioural strategies, such as stimulus control procedures, problem solving training, stress management skills, development of healthy eating habits, assertiveness training, facilitation of social supports, cognitive restructuring of negative maladaptive thoughts and relapse prevention training.	
						**Maintenance phase (12 mo.):**	
						- Recommendation of endurance and resistance training: 5x30–40 min/wk.	
						- Nutritional education and a table listing the energy content of popular foods was provided and explained	
						-Patients were strongly encouraged to contact dieticians, therapists and psychologists for counselling at any time	
Hofso [47] Norway	63 (70)	47.0±11.0	43.3±5.0	12	3	**Intermittent program** (1wk. center, 10 wk. home, 4wk. center, 12wk home, 1wk. center, 23 wk. home, 1 wk. center):	
						- Organized PA sessions: 5x180–240 min/wk	
						.- General dietary recommendation	
						- Motivational interview: client-centred counselling that aims to invoke behavioral changes. Group sessions on emotional aspects of sedentary behavior. Classroom lessons on topics related to PA, nutrition, and co-morbidities.	
						- At home: follow-up by phone to encourage to self-monitor their eating habits and physical activities.	
Hofso [46] *Subjects without diabetes with normal or abnormal glucose tolerance* Norway	33 (73)	43.2±11.6	43.3±5.2	12	3	- Idem Hofso et al. [47]	
	22 (68)	51.3±8.1	43.5 ±4.5				
Maehlum [87] Norway	166 (69)	42.1±10.6	45.7±8.6	12	3	**Intermittent program** (14 wk. center, 16 wk. home, 1 wk. center, 15 wk. home, 1 wk. center, 5 wk. home):	
						- Low-to-moderate intensity endurance exercise sessions and resistance training in group: 5x135 min/wk. using a heart rate watch.	
						- Low calorie diet supported by lectures on nutrition and cooking classes.	
						- Coping and motivational strategies including relationship to food, ability to stick to a plan, and interrelation with others.	
						- At home: follow-up by email or phone according to a structured plan.	
Merrill [35] USA	480 (Not provided)	Not provided	Not provided	12	Not provided	**Telephone-based intervention:**	
						- Recommendation of moderate-intense PA most days of the wk. Pedometer and log to report steps were provided.	
						- Recommendation to reduce total calories, supported with general information on nutrition	
						- Goal setting: identifying emotional eating triggers and changing eating patterns, learning to read food labels, increasing the amount of water consumed, keeping track of food and beverage intake, eating five or six small meals and snacks a day, learning to control portion sizes, adding more fruits and vegetables to the diet, increasing whole-grains, developing a realistic program of regular PA, building and maintaining a support system fora healthy lifestyle, choosing healthy snacks and desserts, choosing healthy beverages, and learning to lower the amount of fat in the diet. Educational workbook was provided. Books, tip sheets, and articles were available if needed.	
Ramani [32] *Subjects with advanced systolic heart failure* USA	10 (60)	45.0±9.0	47.2±3.6	12	Not provided	- Standard recommendations on calorie-restricted diet and exercise.	
Roffey [63] *Low back pain subjects* Canada	46 (80)	50.1±12.9	44.7±7.6	12	3	**Weight-loss phase (6 mo.):**	
						- Diet: 900 kcal/d with 4 provided meal replacements for 12 wk.; the next 3 wk. from 4 to 0 meal replacement; then, diet of 1200–1500 kcal/d.	
						**Maintenance phase (6 mo):**	
						- PA recommendation of 5x60–90 min/wk.	
						- Diet: 1200–1500 kcal/d.	
						- Monthly group sessions on the importance of engaging in additional daily PA and maintaining motivation levels necessary to prevent relapse into previously harmful behaviors.	
Unick [64] *Subjects with type 2 diabetes* USA	654 class II (62) 562 class III (66)	58.4±6.6	37.4±1.5	12	3	- Home-based PA plan ≤ 175 min/wk. at moderate intensity. Pedometer provided.	
						- Diet: 1200–1800 kcal/d, replacing 2 meals and 1 snack with provided meal replacements during 4 mo. Then, only 1 meal and 1 snack were replaced.	
		56.4±6.4	44.8±3.9			- Individual and group sessions on behavioral strategies to help participants achieve their diet and exercise goals, stressing daily self-monitoring of diet and PA: goal-setting, stimulus control, and problem solving. If needed, advanced behavioral strategies such as motivational interviewing and problem-solving techniques were used.	
Konopko-Z. [75] Poland	15 (60)	42.8±9.4	47.1±6.9	11	2	- Physical exercise prescription: 5x45min/wk.	
						- Diet: 1500 kcal/d. Patients recorded the amount and type of foods eaten in specially prepared notebooks, checked once a month.	
Aadland [79] Norway	35 (71)	47.9±8.8	43.2±5.1	10	Not provided	**Intermittent program** (6wk. center, 3–5 mo. home, 4wk. center, 3–5 mo. home, 2wk. center):	
						- Supervised and structured endurance and resistance training: 5x110–150 min/wk. Individualized exercise plan for PA at home. PA training diaries provided.	
						- Recommendation to reduce total calories, based on general information on nutrition	
						- Cognitive behavioral therapy (no more details available)	
Yoshida [80] Japan	18 (100)	41.2±9.2	42.2±3.7	7	Not provided	- PA energy expenditure recommended: 5x300 kcal/wk. with pedometer records	
						- Diet: 940–1100 kcal/d within 4 meal replacements.	
Annesi [66] *Subjects with prehypertension/ hypertension* USA	140 (100)	45.1±9.8	40.4±4.4	6	2	- Recommendation of 150 min/wk. of moderate-intensity endurance exercise with provided access to fitness center.	
						- General dietary recommendation	
						- 12 individual and group meetings of 1h based on social-cognitive and educational methods and self-efficacy theory for exercise and nutrition: orientation to exercise apparatus, self-management/self-regulatory methods ((e.g., long- and short-term goal setting, recording incremental progress, cognitive restructuring, stimulus control, and relapse prevention [preparing for barriers and recovering from lapses] and instruction in skills such as cognitive restructuring, stimulus control, and preparedness for occurrences of barriers to exercise. Goal-setting processes and self-regulatory skills, and development of perceived competence (i.e. self-efficacy) were used. Stress management component composed with deep breathing and muscle relaxation and instructions on appropriate prompts for utilization of these methods were given.	
Annesi [65] *Subjects with prehypertension/ hypertension* USA	155 (100)	44.8±9.8	41.2±5.2	6	2	- Idem, Annesi et al. [66]	
Annesi [71] USA	183 (77)	43.9±9.9	42.0±5.9	6	2	- Idem, Annesi et al. [66]	
Annesi [72] USA	106 (77)	43.5±10	42.0±6.0	6	2	- Idem, Annesi et al. [66]	
Annesi [74] USA	57 (100)	44.2±9.4*	43.6±2.8	6	2	- Idem, Annesi et al. [66] except for PA: individualized endurance training: 3x20–30 min/wk. at 60–70% VO_2_max.	
Annesi [78] USA	57 (100)	44.4±10.3	43.8±2.9	6	2	- Idem, Annesi et al. [74]	
Annesi [73] USA	51 (100)	43.9±9.8	43.8±2.8	6	2	- Idem, Annesi et al. [74]	
Brumley [34] USA	5027 (Not provided)	Not provided	Not provided	6	2	**Telephone-based intervention:**	
						- 10 calls over a 6-to-7 mo. period	
						- Motivational interviewing, behavioral counseling, care management: readiness to change status (Prochaska’s Stages of Change model), assessment of comorbidities, nutritional counseling and tips, exercise recommendations, smoking cessation discussions, review of educational mailing and tools, behavioral change techniques, motivational support, identification of barriers to change, and individualized goals and action plans	
Malone [77] *Prebariatric subjects* USA	19 (74)	40.3±8.8	47.2±4.9	6	2	- Exercise and dietary advices (typically, a reduced calorie, low-fat, low-carbohydrate, high-protein diet), supported by food diary, calorie count guide, and meal planning information	
Fachnie [37] USA	38 (90)	42.0	43.05	4–22	3	- Walking recommendation: ≥3x20–30 min/wk.	
						- Diet: 1000–1200 kcal for 2–3 wk.; then, 420 kcal/d. for 16 wk.; finally 1000–1200 kcal/d. during the maintenance phase (>2wk.)	
						- Dietary therapy: weekly lectures and support sessions to enhance compliance and to educate about healthy and unhealthy eating behaviors	
Oksanen [36] Finland	254 (72)	18–60	45.3±5.5	4	3	- Diet: Optional VLCD period with full meal replacements provided during 6 to 14 wk.; then, 500–1000 kcal/d. and no meal replacement	
						- 16 weekly 60-min group sessions	
						- Behavioral modifications strategies, including dietary and exercise counseling	
Helge [81] Danemark	14 (100)	32.0±11.2	48.0±11.2	3.75	3	- Supervised individual endurance training at moderate intensity:5x120–180min	
	9 (0)	35.0±6.0	49.0±9.0			- Hypocaloric diet: calculate to reduce the body weight by 1% /wk. according to the individual age, body weight and level of PA	
Cancello [84] Italy	8 (63)	48.2±8.7	39.7±5.1	3	3	- Supervised exercise program: 2x60 min/wk.	
						- Dietary recommendation; alimentary diary was weekly reviewed and discussed	
						- Educational group sessions: 2x/wk.	
Benson [76] *Prebariatric subjects* USA	75 (75)	44.1±11.2	46.2±7.0	2.96	2	**Telephone-based intervention:**	
						- Exercise recommendation of ≥ 30 min of mild-to-moderate PA intensity most days of the wk.	
						- Program manual focused on nutrition and exercise strategies	
						Behavioral change support tools (pedometer, food/activity log, coaching call on problem solving and supportive feedback on progress)	
						- Health-Coaching Topics: (1) explanation of the course to help the participant anticipate postsurgery change and long-term success.	
						(2) information on the types of surgical procedures (risks and benefits of weight loss), and helps to prepare lifestyle changes to ensure long-term success.	
						(3) support to create a plan with specific tactics including daily breakfast, portion size control, and journaling (self-monitoring).	
						(4) discussion of recommended levels of PA and benefits, introduces the use of a pedometer, kinds of PA that the participant can initiate and enjoy.	
						(5) impact of common stressors (such as financial problems, conflict with family/friends, job-related issues, etc.) on weight and weight-related behaviors.	
						(6) addressing strategies that help the participant in making progress despite challenges: anticipation of risky situations, how to deal with lapses and relapses, how to engage in problem solving, and seeking support to get through tough situations.	
						(7) recognition that eating in response to specific feelings is emotional eating and interferes with weight management.	
						(8) definition of exercise, various types of exercise, and a discussion on what exercise can do for weight management and feeling energized.	
						(9) addressing how self-talk can direct actions, assess performance, simplify decision making, set patterns and routines, determine which options to consider, helpmeet challenges, or help make lifestyle changes.	
Carlin [39] *Prebariatric subjects* USA	295 (89)	45.0±10.0	51.0±7.0	≈ 2	Not provided	- Individually tailored exercise program	
						- Diet: any earlier successful diet for weight loss was allowed	
						- Dietary recommendation	
Huerta [83] *Prebariatric subjects* USA	5 (0)	54.7±5.8	64.3±4.7	2.7	3	- Water-based exercise program: 1–2x30min /wk.	
						- Diet: < 891 kcal/d. within 6 meal replacements and supplements	
Bader [61] USA	42 (50)	51.0±11.0	46.3±5.8	2.5	3	- Endurance training: 3x30–40 min/wk. at 45–85% heart rate reserve = 1000–2000 kcal/wk.	
						- Diet: 500 kcal deficit /d.	
						- Weekly group nutritional counseling sessions to review dietary information and behavioral strategies. Smoking cessation counseling was provided	
Cuntz [70] Germany	109 (84)	37.1±10.8	44.8±8.7	2.5	Not provided	- Physical exercises and fitness training	
						- Nutritional education	
						- Cognitive behavioral therapy, regulation of eating behavior, social skills training	
Valderas [60] Chile	8 (50)	32.1±9.3	39.1±4.8	2	3	- Endurance and resistance training: 180 min/wk.	
						- Diet: 1300–1800 kcal/d.	
						- Daily care program including behavioral modification	
Formiguera [82] Spain	65 (82)	45.0±7.2	43.0±7.0	1.5	3	- Unscheduled resistance training (hygiene of the column, and development of the abdominal musculature) + light ambulation exercise 2h/day	
						- Very low caloric diet: 399 kcal/meal within 3 provided meal replacements (Modifast Multidiet)	
Gondoni [67] *Subjects with hypertension* Italy	40 (70)	53.0±11.3	42.9±5.8	1.1	3	- Endurance training: 6x60–120 min/wk. at 3–4 METs	
						- Diet: 1463±194 kcal/d.	
Clini [62] *Subjects with sleep-disturbance* Italy	59 (70)	60.0±10.0	47.0±8.0	1	3	- Resistance training and supervised incremental endurance training 10–30 min up to 70–80% of the max load on cycloergometer	
						- Written advice on how to maintain physical fitness was given to patients upon discharge	
						- Diet: LCD supported with sessions of nutritional education 2x/wk.	
						- Behavioral therapy sessions 2x/wk. on self-control, cognitive restructuring reinforcement and relapse prevention	
Facchini [108] Italy	40 (75)	30.0±7.0	41.4±4.6	0.75	3	- Idem, Sartorio [85]	
Morpurgo [109] Italy	10 (70)	35.0±9.3	45.2±10.6	0.75	3	- Idem, Sartorio [85]	
Sartorio [89] Italy	71 (75)	29.3±6.7	41.3±4.2	0.75	3	- Idem, Sartorio [85]	
Sartorio [110] Italy	200 (80)	49.7±14.1	42.7±5.7	0.75	3	- Idem, Sartorio [85]	
Sartorio [85] Italy	28 (0)	29.2±6.9	41.3±4.0	0.75	3	- Idem, Maffiuletti [69], except for supervised aerobic and strength training: 5x35 min/wk. at 50–60% VO_2_max + 5x(1x15 repetitions)/wk at 40–60% 1RM.
	67 (100)	29.4±7.1	41.1±4.1			
Sartorio [88] Italy	54 (70)	29.8±7.3	41.8±0.7	0.75	3	- Idem, Sartorio [85]	
Sartorio [111] Italy	8 (100)	66.5±4.1	38.9±2.6	0.75	3	- Idem, Sartorio [85] except for the diet: 1100–1500 kcal/d.	
Sartorio [86] Italy	60 (68)	(18–68)	40.8±4.8	0.75	3	- Aerobic PA training at 50–60% VO_2_max: 5x60 min/wk + walking 5x3–4 km/wk.	
						- Diet: 1200–1500 kcal/d.	
						- Individual or group psychological counseling: 2–3x/wk.	
Ahmadi [33] USA	14 (50)	32.0	(Not provided)	(0.21–3)	Not provided	- Half supervised exercise: 3.3±2.1 h/d.	
						- Lectures on exercise combined with exercise journal	
						- Energy-restricted diet supported by general nutritional education and daily food journal and never <70% of their resting daily energy expenditure	

* = baseline age of the overall baseline population;

CCT = clinical controlled study; d. = day; HR = heart rate; mo. = month; LCD = low caloric diet; OSA = obstructive sleep apnea; PA = physical activity; RCT = randomized controlled study; RM = repetition maximal; VLCD = very low calories diet; VO_2_max = maximal oxygen consumption; wk. = week

Briefly, 4 studies (7%) with a real control group [48–51], 7 (13%) studies with multiple groups comparing different intervention modalities [52–58] and 44 (80%) uncontrolled studies were included. Nearly half of the studies (45%; n = 25) were published after 2010. Most studies were conducted in the United States (40%; n = 22) and Italy (27%; n = 15). All of the 55 studies assessed body weight, followed by cardiometabolic risk factors (51%; n = 28), PA (27%; n = 15) and waist circumference (22%; n = 12). Nutritional behaviors (15%; n = 8) and quality of life (4%; n = 2) were the least studied outcomes.

### Population

The sample size was small in general: between 5 and 50 subjects in 22 studies (40%), 51–100 in 18 studies (33%), and >100 in 15 studies (27%). The mean age of subjects ranged between 29 and 66.5 years. In 37 studies (67%) mean age was > 40 years. Four studies (7%) were composed of ≤ 50% of women [33,59–61] and all others had a majority of women (76%; n = 42)(missing data 4%, n = 2 [34,35]). In addition, 8 studies (15%) included only class II and III obese individuals with specific comorbidities (i.e, obstructive sleep apnea [59], sleep-disturbance related symptoms and disabilities [62], low back pain [63], type 2 diabetes [64], prehypertension/hypertension [65–67], or advanced systolic heart failure [32]).

### Intervention modalities

The intervention lengths varied from 1–3 weeks to 61.5 months. Twenty-six studies (47%) proposed short-term interventions and 14 (25%) long-term interventions. A maintenance phase was part of the intervention among 6 studies (11%) [37,38,53,63,68,69], ranging from 4 to 60 months after the end of the weight loss phase. An observational follow-up lasting between 4 to 18 months was proposed in only 4 studies (7%) [33,54,62,70]. The majority of studies (n = 32; 58%) had a high-frequency of contact, 13 (24%) a moderate [34,50,58,65,66,71–78], one (2%) low [59], not reported in 9 (16%) [32,33,35,39,51,53,70,79,80].

Supervised or semi-supervised exercise sessions (51%; n = 28) were more frequently used than non-supervised exercise program (33%; n = 18) and PA recommendation (20%; n = 11). In 38 studies (69%), subjects had a caloric restriction ranging from 412 kcal/d to 2190 kcal/d. Only 11 studies (20%) did not have BT programs [32,33,37,39,67,75,77,80–83].

Seventeen studies (31%) used material to support the interventions. Three studies (5%) were telephone-based interventions [34,35,76]. Four studies (7%) [59,75,80,81] proposed only individual face to face, four (7%) only group meeting [33,36,63,84], two (4%) combined individual, group and telephone contacts [47,55], and all others (56%; n = 31) combined individual and group interventions (no data for 11 studies (11%)).

One to five health professionals were involved in the interventions. Dietician was the most common health professional (62%; n = 34), followed by exercise specialist (56%; n = 31), practitioner (47%; n = 26), psychologist (38%; n = 21), nurse (16%; n = 9), occupational therapist or physiotherapist (2%, n = 1) [52], and social workers (2%; n = 1) [52].

### Quality assessment

Inter-rater reliability for quality was high with a Cohen’s kappa coefficient of 0.85. Only four studies (7%) were rated as high quality studies [52,54,55,57], one was weak (2%) [32], and all others were of moderate quality (91%; n = 50) (S1 Table). The outcome assessors were blinded to the intervention or exposure status of participants in only four studies [51,55,59,77]. Tools to assess anthropometric, cardiometabolic risk factors and quality of life were shown as valid and reliable in the vast majority of studies (93%; n = 51). In most studies assessing PA changes (80%; n = 12/15), PA level was self-reported and only three studies used pedometer or accelerometer [48,55,80]. The percentage of participants completing the study was not mentioned in 22% of the studies (n = 12) and below 60% in 7% (n = 4). Only 22% of the studies performed an intention-to-treat analysis (n = 12) [35,50,52,55,58,59,66,71–74,78].

### Effects of lifestyle interventions on weight loss, anthropometric and cardiometabolic risk factors

#### Meta-analysis

The analysis showed significant moderate to high degree of heterogeneity among all included studies. A significant global effect of lifestyle interventions was found on all outcomes studied (p<0.001), except for HDL-C and fasting blood glucose.

**Fig 2:**
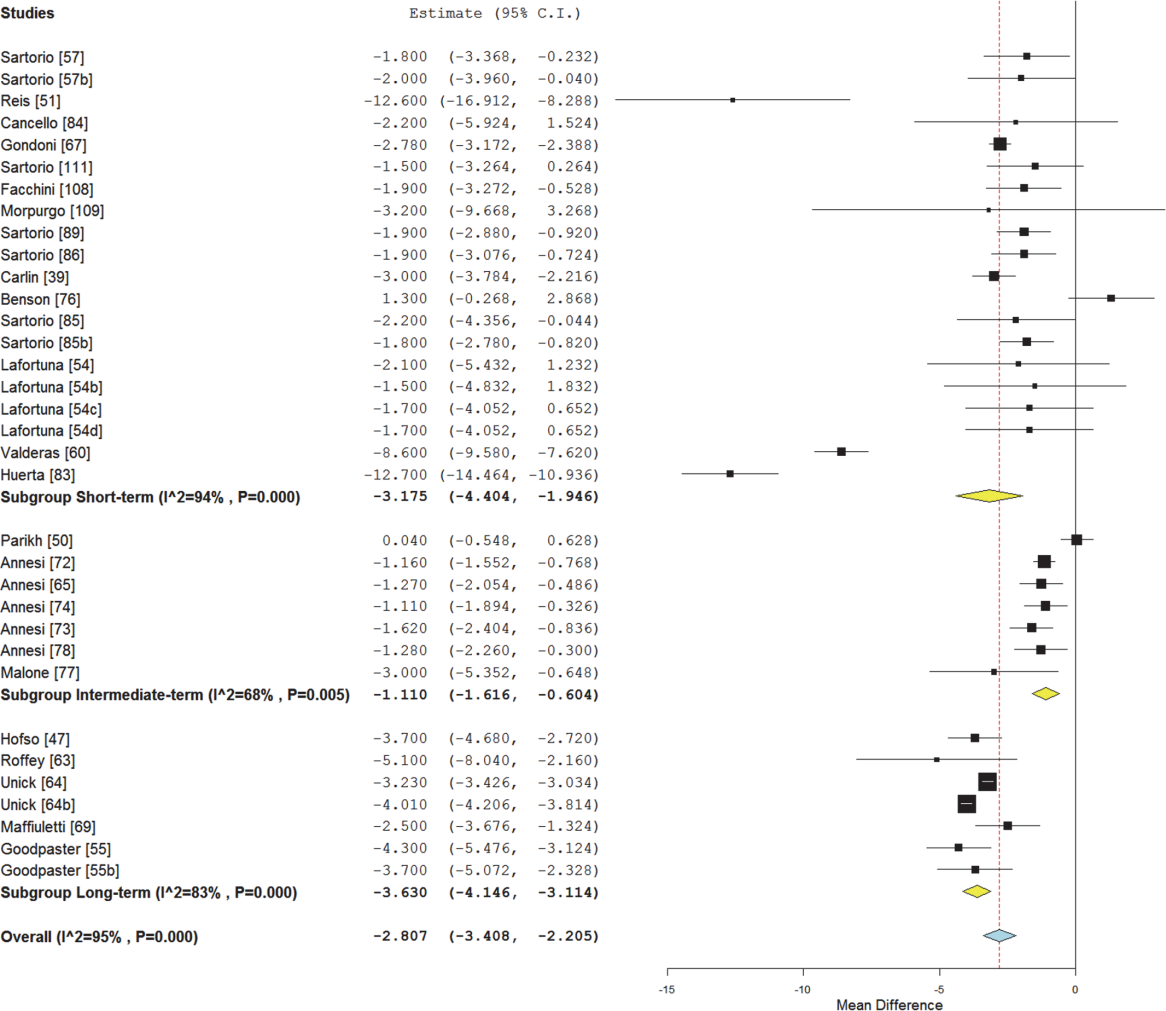
Forest plot of mean body mass index changes according to the intervention length in class II and III obese individuals.

**Fig 3:**
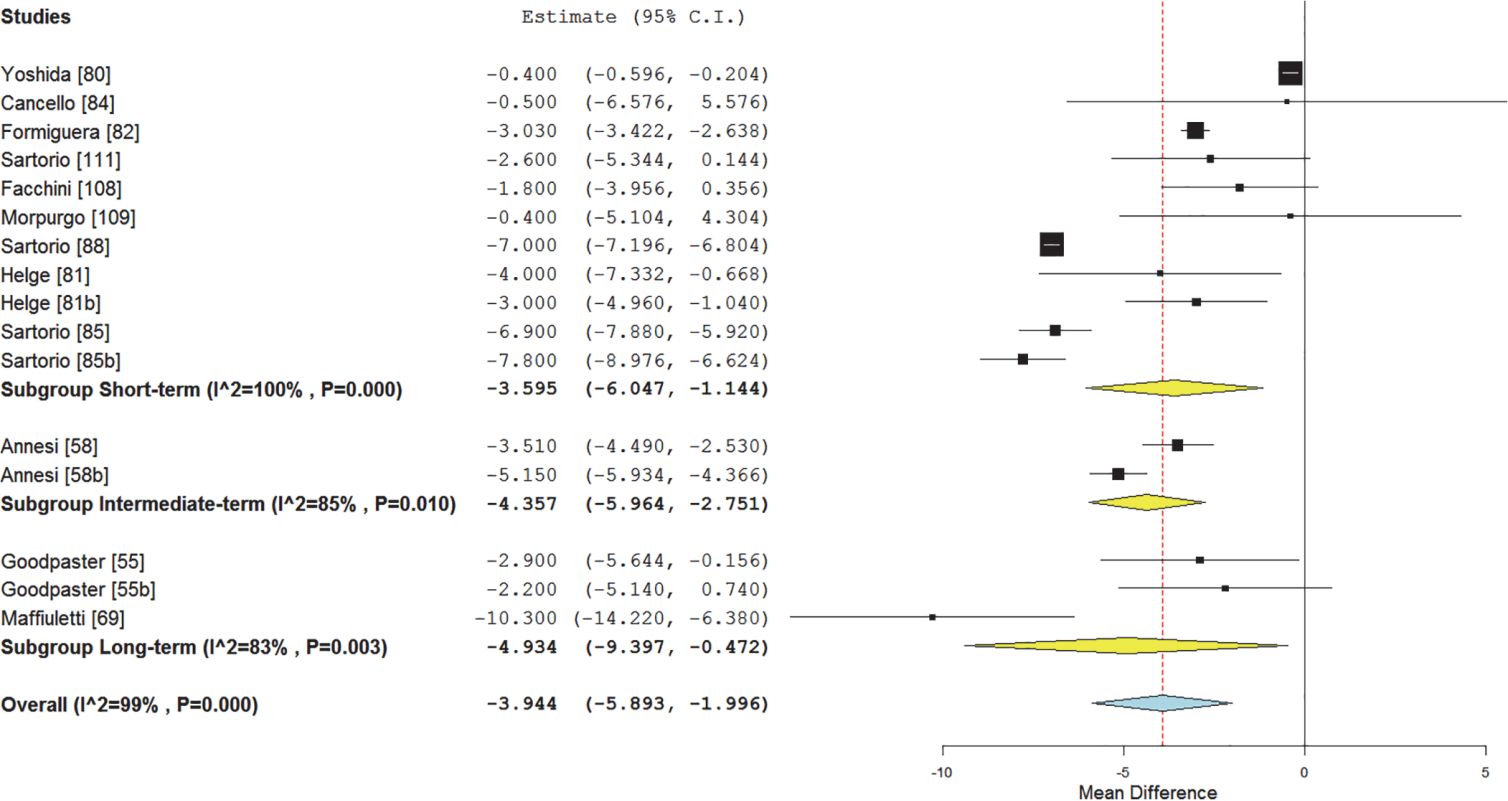
Forest plot of mean fat mass changes according to the intervention length in class II and III obese individuals.

**Fig 4:**
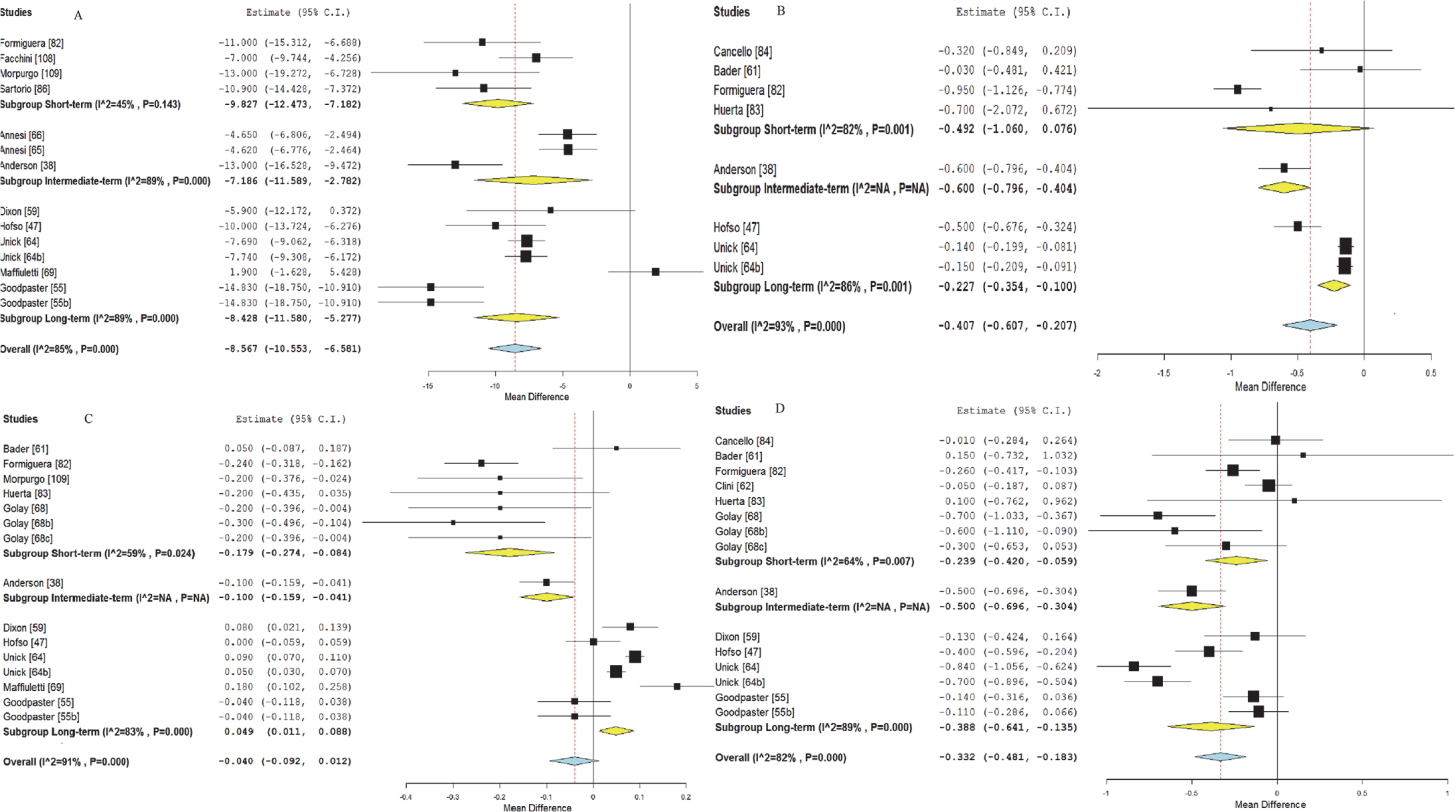
Forest plot of mean systolic blood pressure, LDL cholesterol, HDL cholesterol and triglycerides differences according to the intervention length in class II and III obese individuals. Notes: A (upper corner left): systolic blood pressure; B (upper corner right): LDL cholesterol; C: HDL cholesterol; D: Triglycerides. Letters inserted with the references (b, c, d) represent the different arms of intervention from the same study. A description of each intervention is given in Table 1

Figs. 2 and 3 illustrates mean BMI and fat mass changes according to the intervention length categories, and Fig. 4, mean systolic blood pressure, LDL-C, HDL-C and triglycerides differences.

When studies were separated according to the length of interventions, a significant effect was found on weight loss for short-term (−7.20 kg, 95% CI [−8.88; −5.53], p < 0.01; I² = 94%), intermediate-term (−7.96 kg, 95% CI [−10.82; −5.09], p < 0.01; I² = 97%) and long-term (−11.33 kg, 95% CI [−13.07; −9.59], p < 0.01; I² = 90%) studies (S1 Fig.).

A significant decrease of waist circumference over time was also found for short-term (−4.78 cm; 95% CI [−8.01; −1.55], p = 0.004), intermediate-term (−6.26 cm; 95% CI [−11.82; −0.70], p < 0.01; I² = 90%) and long-term (−7.52 cm; 95% CI [−9.42; −5.61], p < 0.01; I² = 90%) studies (S2 Fig.).

A significant effect on total cholesterol was found only for short-term studies (−0.99; 95% CI [−01.17; −0.81], p < 0.01) (S3 Fig.). Significant reductions of diastolic blood pressure (DBP) were found for short-term (−4.64; 95% CI [−6.71; −2.57], p = 0.05; I² = 61%), intermediate-term (−5.79; 95% CI [−9.14; −2.44], p < 0.01; I² = 93%) and long-term (−3.96; −4.90; −3.03], p = 0.04; I² = 58%) interventions (S3 Fig.). A decrease in fasting glucose was observed only for short-term (−0.53; 95% CI [−0.83; −0.24], p < 0.01; I² = 70%) interventions (S3 Fig.). The subgroup analysis also showed a significant effect on fasting insulin for long-term (−34.77, 95% CI [−47.68; −21.86], p < 0.001; I² = 46%) studies (S3 Fig.).

#### Meta-regression

As shown by meta-regression analyses (S2 Table), intervention length was negatively associated with mean weight loss (S4 Fig.) and waist circumference changes, but positively with SBP, total cholesterol, HDL-C, LDL-C, and fasting glucose changes (p ≤ 0.01). The frequency of contact was negatively associated with weight, BMI, waist circumference, and SBP changes (p ≤ 0.003). Age at inclusion was positively related to fat mass change and negatively with SBP, HDL-C, and fasting glucose changes (p ≤ 0.01). Body weight was positively associated with DBP (p = 0.002).

### Effects of lifestyle interventions on behaviors

Fifteen studies of high and moderate quality assessed the effect of interventions on PA level and 8 on nutritional behavior changes. No meta-analysis was carried out given the heterogeneity of outcome assessment tools (questionnaire, interview, diary, pedometer) and reported data units (kcal/week, steps/day, categorical data, minutes, METs, fruits/vegetables consumed per day). Nine uncontrolled studies and one high quality RCT found significant positive impacts of 6 to 12-month lifestyle interventions on PA level [38,55,58,63–65,69,71,72,76], and five uncontrolled studies on nutritional behaviors [47,58,65,71,76]. However, one RCT showed that the increase in steps/day after 4.5 months of intervention was not significantly different between the walking promotion group meetings and standard support groups (p = 0.46) [48]. In addition, a second RCT found no significant difference in PA level and eating behaviors between the preoperative medically supervised weight management program and the usual care groups [50]. Another study found no significant change in exercise level (KJ/day) and a compensatory increase in energy intake after 4 (+1004 kJ/day) and 7 (+836 kJ/day) months of intervention compared to the data obtained after the first month of intervention [80].

### Effects of lifestyle interventions on quality of life

Only two studies of moderate quality assessed quality of life. The first study showed that a 2-year weight loss program improved significantly 3 dimensions of quality of life: physical function (+12.8%), body pain (+7.2%), and general health scores (+11.6%) as measured with the Short Form-36 Health Survey in 30 class II and III obese individuals with obstructive sleep apnea [59]. The second study reported significant improvements after 1-month of interdisciplinary rehabilitation program in several important quality of life aspects like: sleep, dietary behavior, resistance to fatigue, mobility, activity, mood, emotions, participation, and self-control (Sat-P questionnaire) among 59 class II and III obese participants with sleep-disturbance related symptoms and disabilities [62].

### Effects of sex, age, severity of obesity and metabolic disorders on the effectiveness of lifestyle interventions

Eleven articles provided comparison between females and males. Three studies showed that males lost higher amount of their initial body weight than females after a same short-length lifestyle interventions [53,85,86] without differences in blood pressure change [86].

However, over the 12-month period of lifestyle intervention, 2 studies reported better weight loss in females than males [53,69]. Other studies found no significant sex difference in body weight, waist circumference, and fat mass changes after interventions lasting from 3.75 to 12 months [39,54,79,81,87–89].

Five studies have looked at the effect of age on the effectiveness of lifestyle intervention. Two studies did not identify any association between age and weight loss [39,88] and two others found no significant age difference between the regain and the weight loss groups [68,69]. Another study suggested that older age predicted greater systolic blood pressure improvement after 6-month of lifestyle intervention [66].

Eleven studies were interested in the impact of the severity of obesity on the effectiveness of lifestyle interventions. Seven studies found that subjects with higher initial BMI lost significantly more weight after interventions ranging from 2 to 61.5 months [35,39,55,68,74,79,87]. In contrast, other studies found no difference or association between baseline BMI class and weight loss. [64,69,70,88]. In addition, Unick et al. [64] showed also similar improvements in LDL-C, triglycerides, blood pressure, fasting glucose, and HbA1c at 12 months between class II and III obese individuals. However, class III obese subjects had smaller increase in HDL-C compared with class II (1.8±6.0 vs. 3.3±7.2; p<0.01).

Two studied provided results in normal or abnormal glucose tolerance subjects [46], and glucose-impaired and unimpaired subgroups [76]. Unfortunately, no statistical analyses were performed to compare changes between groups.

### Effects of differences in lifestyle intervention modalities

Seven studies (13%) compared different intervention modalities [52–58].

Martins et al. [52] found that a residential intermittent program and a commercial weight loss camp resulted in greater weight loss compared to a hospital outpatient program (22±13 vs. 18±12 kg vs. 7±10 kg; p < 0.001).

Goodpaster et al. [55] concluded that initial PA intervention had a more beneficial effect on body weight (−10.9 vs. −8.2 kg; p = 0.02), waist circumference (−8.6 vs. −5.2 cm; p = 0.01), and body fat (−8.7 vs. −5.9 kg; p = 0.008) than the 6-month delayed PA practice without significant additional effect on cardiometabolic risk factors.

Three studies compared different supervised exercise modalities combined with diet and BT throughout 12 weeks of intervention. No significant weight loss difference was found between individualized compared to non-individualized training groups [54], and endurance exercise compared to strength and endurance exercise groups [57]. However, endurance and strength exercise training led to greater weight loss (−5.4 vs. −4.0 kg; p < 0.05) compared to non-individualized endurance and resistance exercise training. No significant weight loss difference was found between endurance training and these two groups in the study of Sartorio et al. [56].

Annesi et al. [58] showed that the weight loss and waist circumference reduction was significantly greater in the cognitive-behavioral nutrition intervention than in the nutritional education program (−3.5±6.6 vs. −2.5±4.4 kg; −5.3±5.8 vs. −3.5±6.6 cm).

### Effects of lifestyle interventions on the long-term (follow-up)

Observational follow-up without intervention was performed only in 4 studies [33,54,62,70].

The first study showed no overall significant additional weight loss (Table 1) during the 18-month follow-up period [70]. Nevertheless, 33.6% of subjects continued to lose weight by more than two BMI-points, 29.1% regained weight by more than two BMI-points and 37.3% maintained stable weight [70]. The second study also found an overall weight loss maintenance after the 6-month follow-up period, with 46% of subjects reducing body weight (1 to 5%), 51% regaining and 3% maintaining weight loss (< 1% change) [62].

Lafortuna et al. [54] reported that, after 6 months of follow-up, the individualized 3-week lifestyle group had a higher level of PA compared to the non-individualized group (p<0.05), displaying a trend for further decrease in body weight [54]. Another study provided results from the “Biggest Loser” telecast, where subjects were initially housed together until voted off by their peers every 6–11 days until all were home at 3 months. At 7-months follow-up, the intervention resulted in major reductions in body weight (−39%), body fat (−66%), serum insulin level (−52%), glucose (−21%), and HbA1c (−11%) [33].

Quality of life improvement observed during the first month of interdisciplinary rehabilitation turned down to baseline at 6 months of follow-up. However, the item scores dealing with sleep efficiency, problem solving and social interactions were still maintained at the end of the follow-up period [62].

## Discussion

### Summary of evidences

From the 56 studies included in this review, the majority used uncontrolled design, and most of them were performed after 2010 mainly in women. The analytical part of this present review underlined that most lifestyle interventions containing a PA component are efficacious in class II and III obese individuals. In fact, significant effects were found for body weight, BMI, fat mass, waist circumference, blood pressure, total cholesterol, LDL-C, triglycerides, and fasting insulin.

The pooled mean difference in weight loss was −8.9 kg and −2.8 kg/m² in BMI. Even though this result can seem modest compared to bariatric surgery (10–15 kg/m^2^, 30–50 kg, 15–30% of weight loss)[16,17,90], a weight loss of 5–10%, which represents a reduction of 2–4 kg/m^2^ of BMI [91], is clearly associated with clinically relevant health benefits on glucose and lipids metabolism, blood pressure and psychosocial outcomes (e.g., mood, quality of life, and body image), as well as a reduction in weight-related comorbidities (diabetes, hypertension, hyperlipidemia sleep apnea, osteoarthritis …) in obese individuals [12,92–96].

Waist circumference, which is associated with visceral adipose tissue content [97] showed a pooled mean decrease of −6.9 cm after lifestyle interventions in class II and III obese individuals. This change is promising and could contribute in part to the improvement shown in the other health factors (blood pressure, total cholesterol, LDL-C, triglycerides, fasting glucose and fasting insulin), given the role of visceral adipose tissue in the metabolic alterations [98].

In accordance with other studies in overweight and obese subjects [99,100], anthropometric outcomes (weight, BMI, fat mass and waist circumference) decrease over time after lifestyle intervention in class II and III obese individuals. For example, the subgroup analysis showed that the higher decrease in weight was found with long-term studies (−11.3 kg) compared to short-term and intermediate-term studies with −7.2 kg, and −8.0 kg respectively.

In contrast, a systematic review in overweight and obese subjects found no relationship between the length of the intervention and the percentage of weight loss [101]. In addition, our meta-regression results showed no linear association between intervention length and BMI, and fat mass in contrast with weight and waist circumference. The number of included studies and the absence of intervention diversity in the category intermediate length interventions (7 studies but only 3 tested interventions) could explain this discrepancy. Furthermore, because of incomplete reporting of anthropometric parameters (weight, BMI, fat mass and waist circumference), not all studies have been included in each meta-analysis explaining varying effects. Thus, it is difficult to conclude on the impact of the intervention length on anthropometrics parameters, probably because other factors can impact the results, as shown in our meta-regressions for contact frequency and age. In addition, the number of other potential predictors of weight loss is large (comorbid conditions, individuals’ obesity history, socioeconomic factors like sex, employment, income, education and social status, individual’s quality of life, psychological factors)[102].

Regarding LDL-C, our meta-regression results showed significant linear decrease over time with short-length interventions displaying larger decreases than longer studies. Therefore, time could be a significant moderator as identified by the meta-regression. Nonetheless, the result from the meta-regression should be tempered because for this studied outcome, there was no significant result for short-term studies. This absence of significant result suggests a high degree of variability and heterogeneity in short-term studies and the presence of confounding variables like the use of medications in long-term studies. This reasoning is justified by the large confidence interval as the I².

Concerning the lipid profile, although an overall effect was found, few studies were available and only one study with an intermediate-length was considered. For triglycerides, although not significant, a trend was found for body weight variation, supporting that improvements in triglycerides levels were more likely due to weight variation rather than study length as it is the case for total cholesterol. In fact, for this outcome long-term studies were associated with no significant change. Again, confounding effects of medication use can influence results.

No significant global effect was found for HDL-C in the meta-analysis, since we observed a significant decrease in short-term studies and a significant increase in long-term studies. In contrast with systolic blood pressure, fasting glucose, total cholesterol, and LDL-C, HDL-C tends to improve over time in the meta-regression, as anthropometric outcomes, with long-length studies having larger increases. It is possible that the different baseline comorbidities, medications or PA intensity between studies may explain this discrepancy. Indeed, a systematic review performed in obese adult concluded that the changes in cardiometabolic risk factors are more likely in subjects with abnormal baseline levels [103]. They also stated that “weight loss, irrespective of patient characteristics and intervention, does not uniformly improve cardiovascular risk factors. However, there is a lack of data to correlate weight loss with effect on markers of cardiovascular risk, as there may be weight loss thresholds” [103]. Weight loss or more exactly visceral fat loss is important, however behaviour changes (physical activity and diet) are also necessary to maintain and improve further cardiometabolic alterations on long-term [93,94,104,105]. In addition, medications have little impact on HDL-C level, thus allowing to better capture the effect of lifestyle modification.

### Limitations

If results from the present review and meta-analysis are interesting, some limitations should be discussed. In fact, in the meta-analysis part, a high degree of heterogeneity was detected through the included outcomes. This heterogeneity can be explained by methodological aspects that were not considered in the meta-regression. A second limitation is the fact that we did not use control groups because of the limited number of controlled studies in the literature. This absence of control groups has probably overestimated the results. Third, some papers with relevant data may have been excluded because of missing BMI data, or lack of response from authors to our queries. Fourth, the lack of specific lifestyle key words in our research strategy may have introduced a selection bias. Finally, it cannot be excluded that publication bias could affect our findings.

### Implications for research

Given the small number of high quality studies (n = 4), additional high quality RCT are necessary to improve the current evidence-based knowledge on the beneficial effects of lifestyle interventions including a PA component in class II and III individuals. To improve clinical interpretation, authors have to provide all data on BMI and % of weight loss.

Studies should also consider outcomes beyond weight loss, such as body composition, metabolic risk factors and quality of life. Indeed, as recommended recently by the European Association for the Study of Obesity, obesity management should more focus on ameliorating or maintaining fat-free mass and decreasing fat mass, manage co-morbidities, and improving quality of life and well-being rather than focus on body weight loss [106].

The assessment of health behaviors (nutrition and PA level) is important to reveal subjects’ compliance and to better understand the implication of each lifestyle change in the results of the intervention. Studies on the effect of lifestyle intervention on health behaviors in class II and III subjects are scarce (27%; n = 15) and equivocal, probably due to the different designs, assessment tools (self-reported vs. objective method) and intervention modalities. Thus, future studies have to report subjects’ adherence to the intervention and behavior changes to improve data quality.

Weight loss maintenance after lifestyle interventions seems to follow different patterns according to each subject [62,70]. Additional studies are needed to follow over the long-term the effects of lifestyle interventions on weight loss and other outcomes (body composition, quality of life…) in class II and III obese subjects, since currently only two studies provided this data [62,70].

Insufficient and equivocal results prevent us to summarize the effect of sex, age and obesity severity on the effectiveness of lifestyle interventions. Comparison between gender, age categories, obesity class, metabolic status, responders and non-responders could be another avenue of research to develop. Indeed, predictors of success have to be studied to help health professionals to identify non-responders and better adapt their intervention, since large weight loss difference can be observed between subjects [107].

Only 7 studies compared different intervention modalities. Thus, future researches should investigate the impact of specific intervention modalities (deliverance, length, contact frequency) to better understand optimal intervention. In addition, the report of effective intervention modalities, health professional types and subjects’ characteristics (age, sex, ethnic group, and comorbidities) has to be improved in the studies to be replicated in clinical practice and improve knowledge transfer.

Finally, studies should also include assessment of implementation outcomes (compliance, adverse outcomes and satisfaction) and cost-effectiveness analyses to help health professionals, healthcare managers and policy makers to support lifestyle intervention implementation.

## Conclusions

Lifestyle intervention is effective to improve health in Class II and III obese individuals. Although bariatric surgery is more effective than lifestyle interventions for the treatment of severe obesity and its comorbidities, some individuals have striking response to lifestyle interventions and the number of surgeries performed is insufficient to treat all severely obese individuals. Therefore, lifestyle programs in the hospital and/or primary-care settings should be developed and supported.

## Supporting Information

S1 PRISMA Checklist The PRISMA checklist. (PDF)

S1 Table Quality assessment results categorized by design in overall studies included. UIS = uncontrolled interventional studies; CCT = controlled clinical trial; RCT = randomized controlled trial.
(PDF)

S2 Table Impacts of length, sample size, age of participants, intensity of contact and weight change on the efficacy of lifestyle intervention in class II and III obese individuals. Note: Meta-regression is interpreted as an analysis of regression; the sign gives the direction of the relation (see S3 Fig.)
(PDF)

S1 Fig Forest plot of mean weight loss according to the intervention length in class II and III obese individuals. (TIF)

S2 Fig Forest plot of mean waist circumference according to the intervention length in class II and III obese individuals. (TIF)

S3 Fig Forest plot of mean diastolic blood pressure, total cholesterol, fasting glucose and insulin changes according to the intervention length in class II and III obese individuals. (TIF)

S4 Fig Regression plot of mean difference on weight loss (kg) with studies length as independent variable in class II and III obese individuals. Note: Each circle represents an estimate.
(TIF)
