# Supplementary material for: Effects of Lifestyle Interventions That Include a Physical Activity Component in Class II and III Obese Individuals: A Systematic Review and Meta-Analysis
Source: PLoS One. 2015 Apr 1;10(4):e0119017. doi: 10.1371/journal.pone.0119017 (PMC4382170; doi:10.1371/journal.pone.0119017)
Supplement: S1 Table — UIS = uncontrolled interventional studies; CCT = controlled clinical trial; RCT = randomized controlled trial. (PDF) [file pone.0119017.s002.pdf]

Table S1.

Reference		Design	Score selection biais	Score study design	Score confounders	Score blinding	Score data collection methods	Score withdrawals and dropouts	Global quality rating
Goodpaster [55]	2010	RCT	High	High	High	Moderate	High	Moderate	High
Lafortuna [54]	2003	RCT	Moderate	High	High	Moderate	High	High	High
Martins [52]	2011	CCT	Moderate	High	High	Moderate	High	High	High
Sartorio [57]	2003	CCT	Moderate	High	High	Moderate	High	High	High
Aadland [79]	2012	UIS	Moderate	Moderate	NA	Moderate	High	Weak	Moderate
Ahmadi [33]	2011	UIS	Moderate	Moderate	NA	Moderate	High	High	Moderate
Anderson [38]	1994	UIS	Moderate	Moderate	NA	Moderate	High	High	Moderate
Annesi [58]	2012	RCT	Moderate	High	High	Moderate	High	Weak	Moderate
Annesi [74]	2010	UIS	Moderate	Moderate	NA	Moderate	High	High	Moderate
Annesi [72]	2010	UIS	Moderate	Moderate	NA	Moderate	High	Moderate	Moderate
Annesi [65]	2012	UIS	Moderate	Moderate	NA	Moderate	High	Weak	Moderate
Annesi [66]	2012	UIS	Moderate	Moderate	NA	Moderate	High	High	Moderate
Annesi [78]	2008	UIS	Moderate	Moderate	NA	Moderate	High	Weak	Moderate
Annesi [73]	2010	UIS	Moderate	Moderate	NA	Moderate	High	Weak	Moderate
Annesi [71]	2011	UIS	Moderate	Moderate	NA	Moderate	High	Moderate	Moderate
Bader [61]	2001	UIS	Moderate	Moderate	NA	Moderate	High	Weak	Moderate
Benson [76]	2011	UIS	Moderate	Moderate	NA	Moderate	Moderate	Moderate	Moderate
Bjorvell [49]	1985	CCT	Moderate	High	High	Moderate	High	Moderate	Moderate
Brumley [34]	2007	UIS	Moderate	Moderate	NA	Moderate	Moderate	Weak	Moderate
Cancello [84]	2012	UIS	Weak	Moderate	NA	Moderate	High	High	Moderate
Carlin [39]	2008	UIS	Moderate	Moderate	NA	Moderate	High	NA	Moderate
Clini [62]	2006	UIS	Moderate	Moderate	NA	Moderate	High	High	Moderate
Cuntz [70]	2001	UIS	Moderate	Moderate	NA	Moderate	High	High	Moderate
Dixon [59]	2012	UIS	Moderate	Moderate	NA	Moderate	High	High	Moderate
Facchini [108]	2003	UIS	Moderate	Moderate	NA	Moderate	High	High	Moderate
Fachnie [37]	1987	UIS	Moderate	Moderate	NA	Moderate	High	Weak	Moderate
Formiguera [82]	1991	UIS	Moderate	Moderate	NA	Moderate	High	High	Moderate
Golay [68]	2004	UIS	Moderate	Moderate	NA	Moderate	High	High	Moderate
Gondoni [67]	2003	UIS	Moderate	Moderate	NA	Moderate	High	High	Moderate
Helge [81]	2011	UIS	Moderate	Moderate	NA	Moderate	High	Weak	Moderate
Hemmingsson [48]	2008	RCT	Moderate	High	Weak	Moderate	High	Moderate	Moderate
Hofso [46]	2011	UIS	High	Moderate	NA	Moderate	High	High	Moderate
Hofso [47]	2010	UIS	Moderate	Moderate	NA	Moderate	High	High	Moderate
Huerta [83]	2010	UIS	Weak	Moderate	NA	Moderate	High	High	Moderate
Konopko-Zubrzycka [75]	2009	UIS	Moderate	Moderate	NA	Moderate	High	Weak	Moderate
Maehlum [87]	2012	UIS	Moderate	Moderate	NA	Moderate	High	Moderate	Moderate
Maffiuletti [69]	2005	UIS	Moderate	Moderate	NA	Moderate	High	High	Moderate
Malone [77]	2012	UIS	Moderate	Moderate	NA	Weak	High	Moderate	Moderate
Merrill [35]	2010	UIS	Moderate	Moderate	NA	Moderate	Moderate	Moderate	Moderate
Morpurgo [109]	2003	UIS	Moderate	Moderate	NA	Moderate	High	Weak	Moderate
Oksanen [36]	1996	UIS	Moderate	Moderate	NA	Moderate	High	Moderate	Moderate
Parikh [50]	2012	RCT	Moderate	High	High	Moderate	High	Weak	Moderate
Reis [51]	2012	RCT	Weak	High	High	Moderate	High	Weak	Moderate
Richman [53]	1992	CCT	Moderate	High	High	Moderate	High	Weak	Moderate

Roffey [63]	2011	UIS	Moderate	Moderate	NA	Moderate	High	High	Moderate
Sartorio [56]	2002	RCT	Moderate	High	High	Moderate	High	Weak	Moderate
Sartorio [88]	2003	UIS	Moderate	Moderate	NA	Moderate	High	High	Moderate
Sartorio [89]	2003	UIS	Moderate	Moderate	NA	Moderate	High	High	Moderate
Sartorio [85]	2005	UIS	Moderate	Moderate	NA	Moderate	High	High	Moderate
Sartorio [86]	2001	UIS	Moderate	Moderate	NA	Moderate	Weak	High	Moderate
Sartorio [110]	2003	UIS	Moderate	Moderate	NA	Moderate	High	High	Moderate
Sartorio [111]	2004	UIS	Moderate	Moderate	NA	Moderate	High	High	Moderate
Unick [64]	2011	UIS	Moderate	Moderate	NA	Moderate	High	High	Moderate
Valderas [60]	2010	UIS	Moderate	Moderate	NA	Moderate	High	Weak	Moderate
Yoshida [80]	1995	UIS	Moderate	Moderate	NA	Moderate	High	High	Moderate
Ramani [32]	2008	UIS	Weak	Moderate	NA	Moderate	Weak	High	Weak
