# Supplementary material for: Effects of Lifestyle Interventions That Include a Physical Activity Component in Class II and III Obese Individuals: A Systematic Review and Meta-Analysis
Source: PLoS One. 2015 Apr 1;10(4):e0119017. doi: 10.1371/journal.pone.0119017 (PMC4382170; doi:10.1371/journal.pone.0119017)
Supplement: S2 Table — Note: Meta-regression is interpreted as an analysis of regression; the sign gives the direction of the relation (see S3 Fig.) (PDF) [file pone.0119017.s003.pdf]

Table S2.

MODERATORS	Coefficient	95% CI	Standard error	P value
**Weight**				
Length	-0.531	-0.89 ; - 0.16	0.185	0.004
Sample size	0.011	-0.003; 0.025	0.007	0.138
Age	-0.129	-0.359; 0.101	0.117	0.271
Contact	-7.692	-11.61; -3.77	1.999	< 0.001
**Body mass index**				
Length	-0.092	-0.316; 0.132	0.114	0.421
Sample size	0.003	-0.003; 0.010	0.003	0.315
Age	-0.069	-0.160; 0.022	0.047	0.139
Contact	-2.959	-4.898; -1.021	0.989	0.003
**Fat mass**				
Length	-0.017	-0.254; 0.219	0.121	0.885
Sample size	-0.026	-0.057; 0.005	0.016	0.099
Age	0.117	0.028; 0.207	0.046	0.010
Contact	-5.144	-10.925; 0.637	2.950	0.081
Weight variation	-0.168	-0.360; 0.025	0.098	0.088
**Waist Circumference**				
Length	-0.832	-1.457; -0.208	0.319	0.009
Sample size	-0.007	-0.024; 0.009	0.008	0.389
Age	0.553	-0.326; 1.431	0.448	0.217
Contact	-8.341	-12.975; -3.706	2.365	< 0.001
Weight variation	-0.145	-0.756; 0.467	0.312	0.643
**Systolic blood pressure**				
Length	0.291	0.066; 0.516	0.115	0.011
Sample size	0.022	0.014; 0.029	0.004	< 0.001
Age	-0.655	-0.872; -0.438	0.111	< 0.001
Contact	-5.386	-7.863; -2.909	1.264	< 0.001
Weight variation	0.054	-0.088; 0.197	0.073	0.454
**Diastolic Blood pressure**				
Length	-0.013	-0.258; 0.233	0.125	0.92
Sample size	0.004	-0.002; 0.009	0.003	0.18
Age	0.052	-0.218; 0.321	0.137	0.70
Contact	-1.251	-2.65; 0.1509	0.71	0.08
Weight variation	0.122	0.045; 0.198	0.039	0.002
**Total Cholesterol**				
Length	0.080	0.042; 0.117	0.019	< 0.001
Sample size	-0.005	-0.014; 0.004	0.005	0.237

Age	-0.012	-0.033; 0.009	0.011	0.280
Contact	0.233	-0.277; 0.742	0.260	0.371
Weight variation	0.009	-0.010; 0.027	0.009	0.351
**High density lipoprotein cholesterol**				
Length	0.012	0.008; 0.015	0.002	< 0.001
Sample size	0.001	0.000; 0.001	0.001	< 0.001
Age	-0.014	-0.018; -0.010	0.002	< 0.001
Contact	-0.035	-0.071; 0.000	0.018	0.052
Weight variation	0.001	-0.001; 0.004	0.001	0.362
**Low density lipoprotein cholesterol**				
Length	0.038	0.013; 0.063	0.013	0.002
Sample size	-0.001	-0.002; 0.001	0.001	0.696
Age	0.048	-0.040; 0.135	0.045	0.284
Weight variation	-0.011	-0.028; 0.007	0.009	0.225
**Triglycerides**				
Length	0.010	-0.007; 0.026	0.009	0.261
Sample size	-0.001	-0.001; 0.001	0.001	0.115
Age	-0.022	-0.059; 0.015	0.019	0.236
Contact	0.025	-0.195; 0.246	0.112	0.822
Weight variation	0.010	-0.001; 0.022	0.006	0.073
**Fasting Glucose**				
Length	0.088	-0.032; 0.144	0.029	0.002
Sample size	-0.009	-0.020; 0.002	0.006	0.122
Age	-0.033	-0.053; -0.012	0.011	0.002
Contact	0.316	-0.323; 0.954	0.326	0.332
Weight variation	0.031	-0.021; 0.083	0.026	0.239
**Fasting insulin**				
Length	-2.979	-9.595; 3.637	3.375	0.378
Sample size	0.417	-0.963; 1.798	0.704	0.553
Age	-1.874	-3.886 ; 0.138	1.026	0.068
Contact	-16.855	-91.741; 58.031	38.208	0.659
Weight variation	0.826	-3.884; 5.535	2.403	0.731
