# Supplementary figures and images for: Effects of Lifestyle Interventions That Include a Physical Activity Component in Class II and III Obese Individuals: A Systematic Review and Meta-Analysis
Source: PLoS One. 2015 Apr 1;10(4):e0119017. doi: 10.1371/journal.pone.0119017 (PMC4382170; doi:10.1371/journal.pone.0119017)

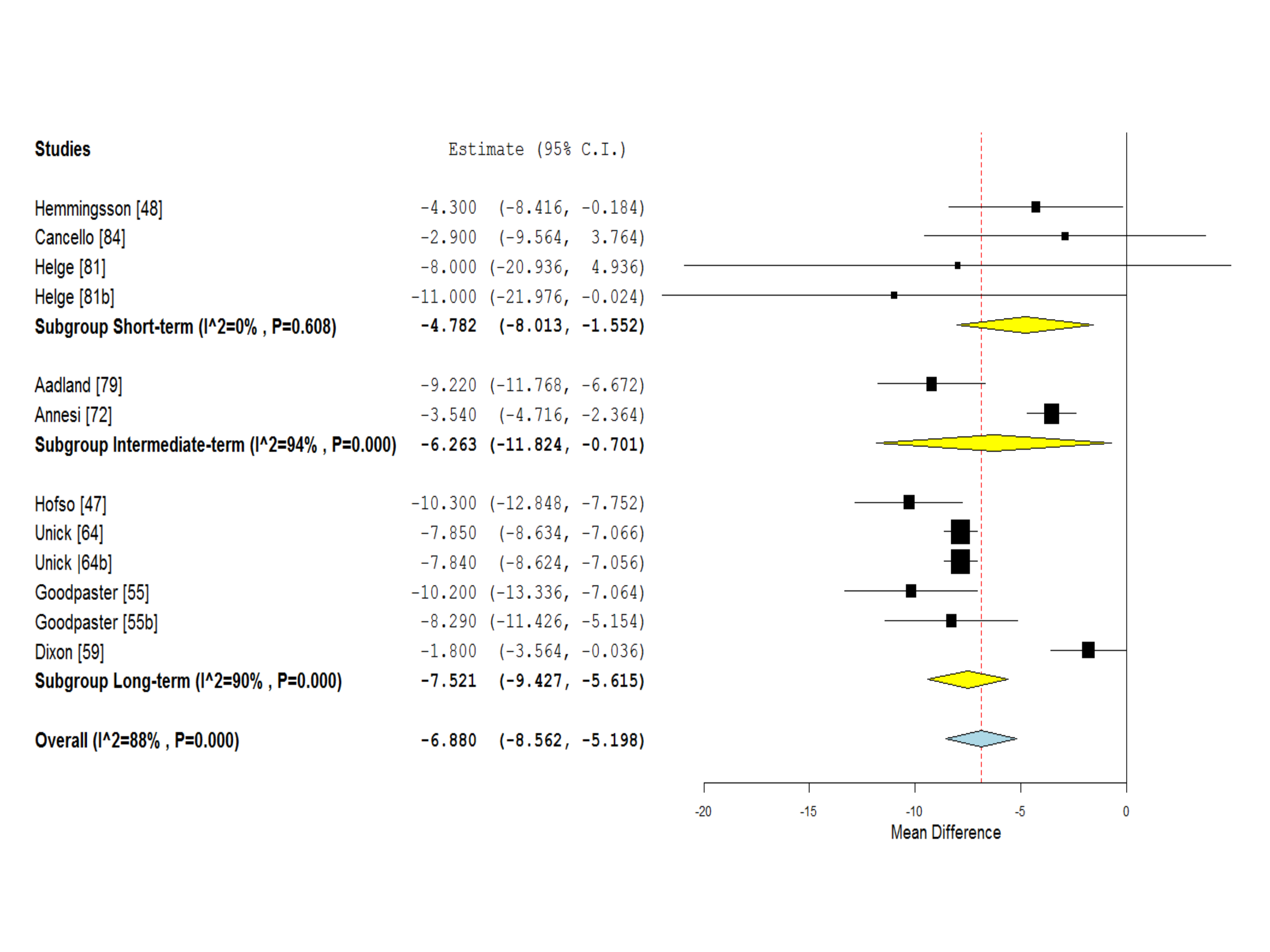

Supplement: S2 Fig — (TIF) [file pone.0119017.s005.tif]

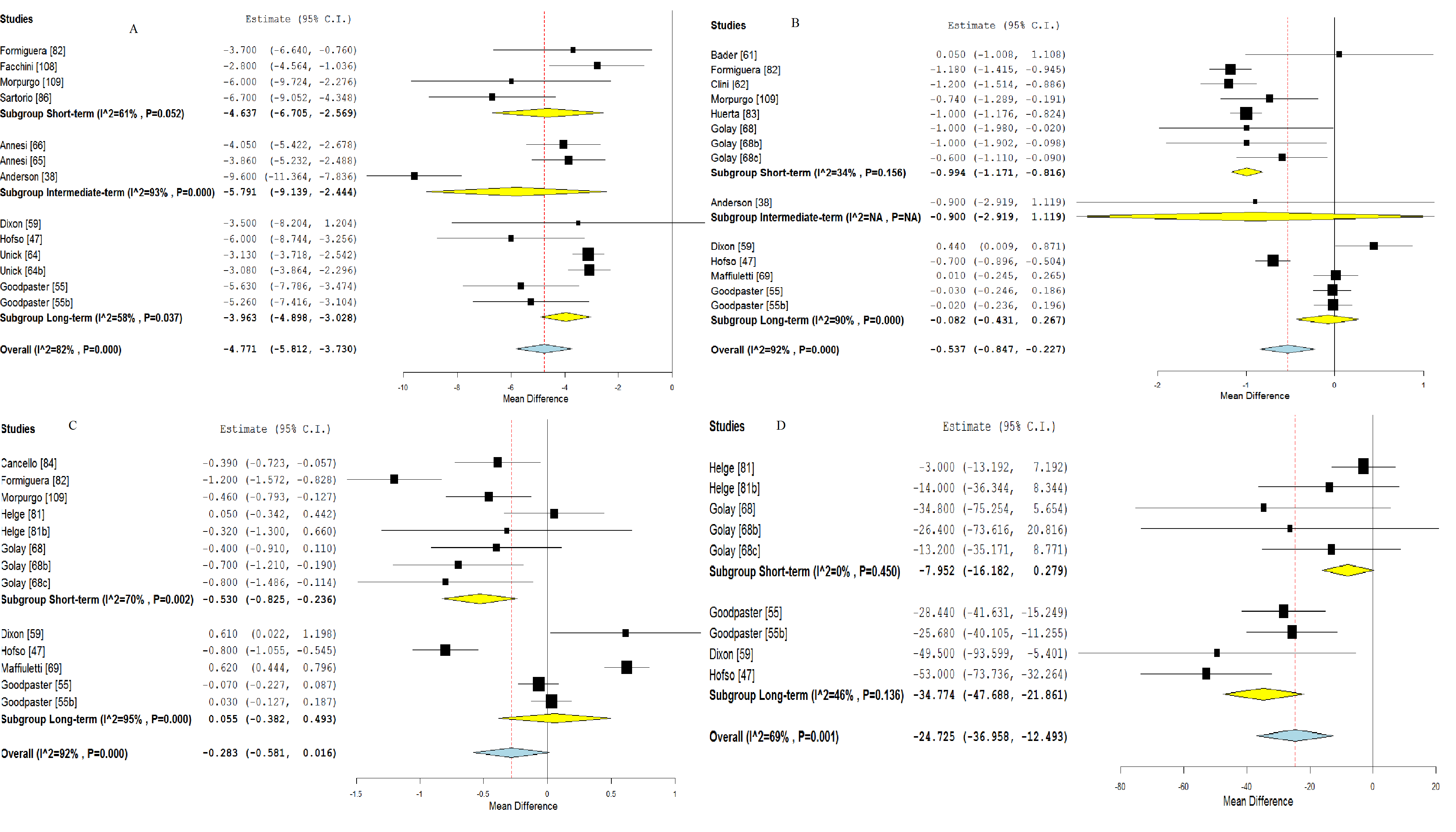

Supplement: S3 Fig — (TIF) [file pone.0119017.s006.tif]

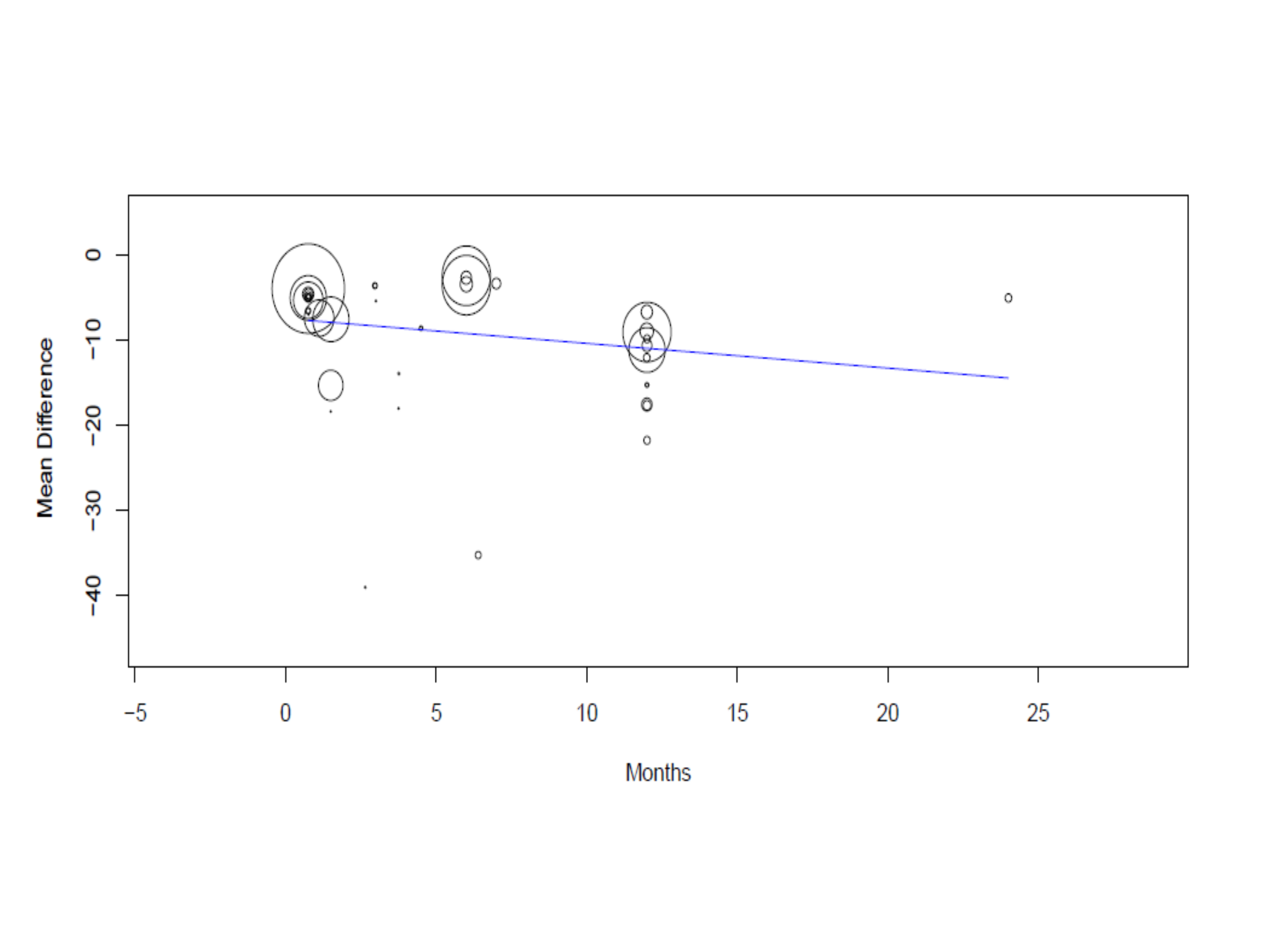

Supplement: S4 Fig — Note: Each circle represents an estimate. (TIF) [file pone.0119017.s007.tif]
